# Recent Publications

**Published:** 1886-02

**Authors:** A. B. Palmer

**Affiliations:** Professor of Pathology, Practice of Medicine and clinical medicine, etc., etc.: Ann Arbor, Md.


### RECENT PUBLICATIONS.

A Treatise on epidemic cholera and allied diseases. By A. B. Palmer, M. D., L.L.D., Professor of Pathology, Practice of Medicine and clinical medicine, etc., etc.: Ann Arbor, Md.

As we are now looking for the cholera to visit us and it can hardly be avoided another season it is well for us to be looking at everything that may be presented on the important subject. The author says in his preface that "Not only the results of the recent investigation of Koch, Emmerich, of Kline, and of many others have been given, but the observations and opinions of those who have encountered the disease in its home in Asia, and as it has appeared in its several migrations over Europe and this continent. The views of various members of the profession of the present time and our own country have been given, of men who have followed the literature of the subject and whose reputation as physicians, medical teachers and writers, entitle them to be heard."

This publication, so timely in view of the probable visitation of Epidemic Cholera, in this country, consists of a volume of about 200 pages, neatly and substantially gotten up, and bound in cloth. It contains a summary of the literature on the subject brought down to the present time, including the recent investigations of Koch and others; and in it the causes, methods of prevention and treatment will be fully discussed, and definite directions will be given. It is sold for $1 a copy and can be obtained of Ann Arbor Register Publishing Co., Ann Arbor, Mich.

The Century for February is equal to its late number. The continual history of the war and the contributions from distinguished actors in that great drama, make a magazine that people who read, will have. The other artists are warm and able.

Bicycle Calendar.—Hail "Columbia!" The Pope Manufacturing Co. have one of the nicest calendars that it has been our fortune to see. Artistic, handsome and useful. Has day of week and month conspicuous on tab in center and a regular cyclopedia on bicycling in quotations. On either side of calendar block is scenery executed in an artistic and appropriate manner. It is ornamental as well as useful.

The Journal of Reconstruction, New York, is a new candidate for pnblic favor and a good one. Published quarterly by Jahnrich, at 50 cents a year.

